# Pharmacokinetic Analysis of Epithelial/Endothelial Cell Barriers in Microfluidic Bilayer Devices with an Air–Liquid Interface

**DOI:** 10.3390/mi11050536

**Published:** 2020-05-25

**Authors:** Timothy S. Frost, Linan Jiang, Yitshak Zohar

**Affiliations:** 1 Department of Biomedical Engineering, University of Arizona, Tucson, AZ 85721, USA; zohar@email.arizona.edu; 2 Department of Aerospace and Mechanical Engineering, University of Arizona, Tucson, AZ 85721, USA; jiangl@email.arizona.edu

**Keywords:** pharmacokinetics, aerosol

## Abstract

As the range of applications of organs-on-chips is broadening, the evaluation of aerosol-based therapies using a lung-on-a-chip model has become an attractive approach. Inhalation therapies are not only minimally invasive but also provide optimal pharmacokinetic conditions for drug absorption. As drug development evolves, it is likely that better screening through use of organs-on-chips can significantly save time and cost. In this work, bio-aerosols of various compounds including insulin were generated using a jet nebulizer. The aerosol flows were driven through microfluidic bilayer devices establishing an air–liquid interface to mimic the blood–air barrier in human small airways. The aerosol flow in the microfluidic devices has been characterized and adjusted to closely match physiological values. The permeability of several compounds, including paracellular and transcellular biomarkers, across epithelial/endothelial cell barriers was measured. Concentration–time plots were established in microfluidic devices with and without cells; the curves were then utilized to extract standard pharmacokinetic parameters such as the area under the curve, maximum concentration, and time to maximum concentration. The cell barrier significantly affected the measured pharmacokinetic parameters, as compound absorption through the barrier decreases with its increasing molecular size. Aerosolizing insulin can lead to the formation of fibrils, prior to its entry to the microfluidic device, with a substantially larger apparent molecular size effectively blocking its paracellular transport. The results demonstrate the advantage of using lung-on-a-chip for drug discovery with applications such as development of novel inhaled therapies.

## 1. Introduction

The development of new compounds to treat diseases, especially cancers, has been a stumbling block in healthcare [1]. In drug discovery and development, pharmacokinetics and pharmacodynamics play an important role contributing to treatment success. Whereas pharmacodynamics is the study of biochemical and physiological effects of a drug on an organism, pharmacokinetics is the study of what an organism does to a drug; both together influence dosing, benefit, and adverse effects [2]. The processes involved in pharmacokinetic analysis—when a drug is taken—include absorption, distribution, metabolism, and excretion (ADME). Historically, new compound research has proven to be highly cost-ineffective, as only 11% of new compounds that enter Phase 1 of clinical trials eventually win approval by the FDA [3]. Indeed, over the last 15 years, the total cost to develop a single new drug has more than doubled [4,5]. However, pharmaceutical research has begun to adopt a smarter, more streamlined methodology to allow for more efficient drug development. This trend is noted as a decrease in the number of new compounds entering early stages of clinical trials, but an increase in the relative percentage of compounds progressing throughout clinical trials [6]. This more streamlined drug discovery strategy includes better compound prioritization at the onset of the drug development process and is aided by the application of more accurate models for drug screening such as organs-on-chips [7]. These plastic microchips provide unique microenvironments for cell co-cultures allowing combinations of multiple cell types to recapitulate the critical functions of human organs [8]. By realizing improved testing grounds for new compounds, less effective compounds can be discarded before launching costly pre-clinical and clinical trials involved in drug development. Indeed, recent estimates claim that using organ-on-a-chip systems to enhance compound selection efficiency may reduce the R&D costs for a new compound by 10–30% [9].

As organ-on-a-chip devices gain more appreciation, the evaluation of aerosol-based therapies utilizing lung-on-a-chip has attracted considerable attention. Indeed, aerosol-based treatments have recently become more widely used to accomplish a variety of tasks. Inhalation therapies have expanded from bronchodilators or antibiotics to more advanced treatments such as systemic gene therapies, needle free vaccinations, and insulin therapy for diabetic patients [10,11,12]. This surge in applications of inhalation therapies is driven by several factors. Inhaled therapies provide optimal pharmacokinetic conditions for a site of drug absorption such as a high surface area, low metabolic and enzymatic activity, and thin air–blood barrier [13,14]. Furthermore, inhalation therapies are minimally invasive and are a preferred method for patients compared to other treatment options [15]. Inhaled insulin is particularly very attractive since it can significantly improve the quality of life of diabetic patients subject to daily injections by providing a non-invasive method for its administration. The formulation of insulin in aerosol form, while extremely exciting, has shown to have serious challenges, such as its structure and toxicity, as seen with the commercial release and eventual termination of Pfizer’s inhalable insulin marketed as Exubera [16]. The potential to test different formulations of aerosol therapies, like insulin, is one of the major benefits of using lung-on-a-chip platforms.

So far, lung-on-a-chip devices have been used in numerous studies to gather new insights into the respiratory system. Several works have focused on modeling different regions of the human respiratory system applying different chip configurations [17,18,19,20]. We previously investigated the spatiotemporal molecular concentration distribution and characterized the permeability of epithelial/endothelial cell co-cultures in microfluidic bilayer devices [21,22]. However, to date, compound absorption characteristics were not analyzed using a true aerosol stream flowing over the modeled epithelium. Aerosol flow in microchannels, like in lung-on-a-chip devices, has been analyzed theoretically and simulated numerically, but has been experimentally tested using only liquid suspensions of particles rather than true aerosols [20]. In other studies, the epical side of epithelial cell cultures grown in Transwells was exposed to aerosol treatments to assess their absorption across the cellular monolayers; however, liquid flow along the basal side of the cells, simulating blood flow, was not incorporated [23,24]. This flow has extensively been shown to influence monolayer absorption characteristics [25,26,27]. The epithelia in lung-on-a-chip systems had been exposed to compounds suspended in an air stream in recent studies, but the work was dedicated to investigating the biological response of the epithelial cells rather than the pharmacokinetic analysis [28,29]. Indeed, the need for absorption studies of compounds transported via an air stream in a lung-on-a-chip has become recognized [30]. In this work, the properties of aerosol flows in microchannels are first documented, and the absorption of aerosolized compounds across epithelial/endothelial cell barriers in microfluidic bilayer devices—mimicking the blood–air barrier—is experimentally characterized.

## 2. Materials and Methods

### 2.1. Experimental Setups

Experiments were conducted under fluid static condition in Transwell inserts and under dynamic flow condition in microfluidic devices.

#### 2.1.1. Transwell Inserts

Commercially-available 12-well Transwells (Corning, Corning, NY, USA), with polyester membranes, were utilized to assess static compound permeability in the absence of flow-induced shear stress. The area of each Transwell membrane was 1.12 cm^2^ with 0.4 µm diameter pores and 0.5% porosity. Epithelial cells were seeded in the Transwell top apical compartment at a density of 2 × 10^6^ cells/mL. Once the cell monolayer had reached confluency, the 500 µL culture media in the top compartment was replaced with a solution of endothelial cell media mixed with various fluorescently-labeled compounds at a pre-determined initial concentration. The bottom basolateral compartment was filled with 1500 µL fresh endothelial cell culture media, and the Transwell was placed in an incubator for 2 h. Then, liquid samples of 100 µL from both compartments were aliquoted into a 96-well plate. Fluorescent intensities of the collected samples were measured, using a BioTek Synergy 2 Plate Reader (BioTek, Winooski, VT, USA), and converted to molecular concentrations as reported elsewhere [21].

#### 2.1.2. Microfluidic Bilayer Devices

**Figure 1:**
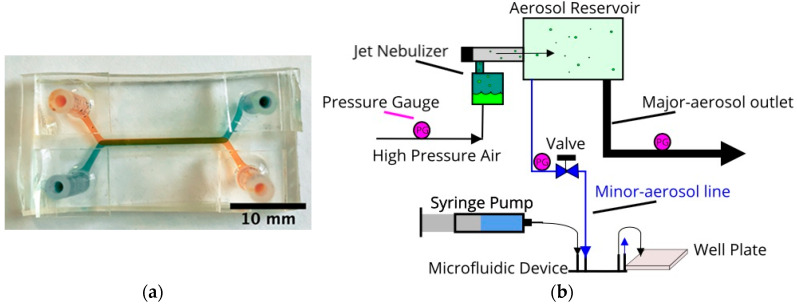
(**a**) A photograph of a fabricated and packaged microfluidic bilayer device with green dye in the top and orange dye in the bottom microchannel. (**b**) A schematic representation of the jet nebulizer setup; the aerosol system is connected to a microdevice’s top channel while the bottom channel is connected to a syringe pump to drive media across the device.

Microfluidic devices, comprised of two stacked microchannels and separated by a porous membrane, were fabricated following soft lithography techniques as previously reported [22]. Briefly, polydimethylsiloxane (PDMS) pre-polymer was poured over an aluminum cast mold and allowed to cure overnight. The cured PDMS substrates with grooves, each about 35 mm long and cross-section area of 500 × 1000 µm, were peeled off the aluminum mold. Semi-permeable polyester membranes, about 20 µm thick and pore diameter of 0.8, 3.0, and 8.0 µm (with porosity of 1%, 10%, and 2.5%, respectively), were treated with a 5% solution of (3-Aminopropyl)triethoxysilane (APTES) to allow for organosilane bonding with the PDMS substrates [31]. Each device was assembled by bonding two microchannels on top of each other, with a treated membrane sandwiched between the overlapping segment about 20 mm in length. The fabrication process was completed with the attachment of tubing adapters over punched holes to serve as inlet/outlet connectors to the external fluid handling system. A photograph of a fabricated and packaged microfluidic bilayer device is shown in Figure 1a.

#### 2.1.3. Aerosol Apparatus

A jet nebulizer (Teleflex, Wayne, PA, USA) was utilized to form liquid droplets and transport them suspended in an air stream, as sketched in Figure 1b [32,33]. Pressurized air driven through a reservoir containing an aqueous fluorescent compound solution emerges as aerosol flow of air stream with liquid droplets. The bulk fluorescent solution was mixed with a common food additive, propylene glycol (<5% v/v), to facilitate stable droplet formation [34]. The aerosol flow, passing through a larger aerosol reservoir, is divided into a major and a minor stream. The minor stream is directed toward the epithelial (top) microchannel, while the endothelial (bottom) microchannel is connected to a syringe pump delivering steady media flow with zero compound concentration; thereby an air–liquid interface is established across the device porous membrane separating the two microchannels. Tracer compound molecules are transported from the aerosol droplets in the epithelial microchannel into the media flow in the endothelial microchannel due to concentration gradients. Samples of the outlet flow from the endothelial microchannel are collected every 2.5 min in a 384-well plate to measure their fluorescent light intensity using a BioTek Plate Reader.

### 2.2. Cell Lines and Tracer Compounds

#### 2.2.1. Epithelial and Endothelial Cell Cultures

**Figure 2:**
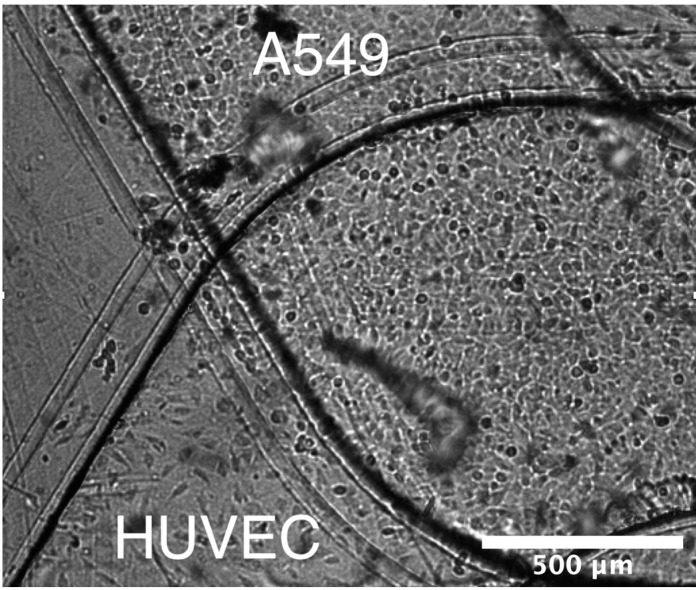
A brightfield image of the two microchannels converging with a monolayer grown in each channel (A549 top channel, HUVEC bottom channel) along the membrane surface.

Epithelial and endothelial cell lines were utilized in order to model the blood–air barrier. A549 pulmonary epithelial cells (ATCC, Manassas, VA, USA) were used to represent the pulmonary epithelium. The A549 cells were cultured using RPMI media with 10% FBS and 1% penicillin streptomycin. Human umbilical vein endothelial cells (HUVEC, ATCC) were used to represent the pulmonary endothelium. The HUVEC cells were cultured using F12K media with 10% fetal bovine serum (FBS), 1% penicillin streptomycin, 150 µg/mL endothelial cell growth supplement (ECGS), and 10 µg/mL heparin. Either epithelial or endothelial cells were seeded in a corresponding microchannel at a density of 2 × 10^6^ cells/mL and, to promote cell adhesion, the microchannels were coated with 200 µg/mL collagen. Once the epithelial and endothelial cell monolayers reached confluency, both cell cultures were maintained in endothelial media flows to allow direct comparison between results obtained in microfluidic devices and Transwells. A brightfield image of the two separate epithelial and endothelial cell cultures within a microfluidic bilayer device is shown in Figure 2.

#### 2.2.2. Fluorescent Tracer Molecules

Fluorescently tagged molecules were selected for pharmacokinetic analysis of similar therapeutic compounds. The initial concentration of each molecule was adjusted based on its intensity vs. concentration calibration curve to allow for reliable signal intensity measurement while avoiding the saturation limit. Fluorescein (33 kDa, 26 µM) was used as a low-cost agent to enable droplet visualization and, subsequently, aerosol characterization. Low molecular weight dextran (LMW Dextran, 4 kDa, 60 µM) was used to model smaller compound transport. A slightly larger anionic dextran (C-Dextran, 10 kDa, 3 µM) was used to assess medium sized compound transport as well as any ionic charge effects. High molecular weight dextran (HMW Dextran, 70 kDa, 1 µM) was used to simulate the absorption of larger therapeutic compounds. Lastly, insulin (5.8 kDa, 3 µM) was used to investigate its potential application as an inhaled therapeutic compound.

#### 2.2.3. Cell Cytotoxicity Evaluation

In general, higher compound concentration is desired since the emitted light intensity increases with molecular concentration enhancing the signal-to-noise ratio and, thereby, improving the reliability of the measured concentration. However, some compounds could be toxic agents to cell cultures; as the signal intensity increases with increasing compound concentration, cells could be adversely affected resulting in cell death [35]. Hence, cellular cytotoxicity was evaluated using a trypan blue cell viability assay [36]. Epithelial and endothelial cells were seeded and cultured in 96-well plates for 48 h. The cell cultures were exposed to the selected compounds at various concentrations for 1 h and then washed using PBS. Next, the wells were immersed in 0.2% Trypan Blue for 10 min, after which the cells were fixed using 4% paraformaldehyde and rinsed with PBS. Using a brightfield microscope, dead cells were counted using uptake of trypan blue as a marker for nonviable cells.

The exposure of both A549 and HUVEC cell lines to fluorescent tracers such as FITC-Dextrans of various sizes did not trigger cellular apoptosis within the tested concentration range of 10^−3^–10^1^ µM. On the other hand, while FITC-Insulin at higher concentration did not have adverse effect on HUVEC endothelial cells, the A549 epithelial cell viability dropped to 88% under exposure to insulin at concentration of 10 µM. This is consistent with previously reported observations that some cell types start to exhibit signs of cell death following 30 min exposure to insulin at concentrations greater than 1 µM [37,38]. Insulin may cause cell death due to its dynamic nature in solutions. Insulin is produced and stored in the body as a hexamer (a unit of six insulin molecules), while the active form is the monomer. The hexamer is an inactive form with long-term stability, which serves as a way to keep the highly reactive insulin protected, yet readily available [39]. Improperly folded insulin; however, can prove destructive to cells. Insulin folded incorrectly can create larger protein structures known as amyloids that are lethal to cells [40]. The formation of these amyloids is highly dependent on the cell membrane characteristics and the interacting proteins expressed there [38]. This could be the reason why the epithelial cell line did experience significant cell death while the endothelial cell line did not.

### 2.3. Aerosol Flow Characterization

Proper scaling for organ-on-a-chip applications is a widely contested issue; it is typically done either following allometric scaling or using a specific kinetic constant such as consumption by specific cell types to scale other parameters [41,42]. The controlled variables in this work, summarized in Table 1, such as the aerosol inlet pressure of 0.1 psig, were adjusted in an effort to closely match reported clinical or physiological values for drug absorption at the alveolar sac level as done in similar studies [28]. Further detailed characteristics of the aerosol flow are available in the Supplemental Information.

## 3. Results and Discussion

### 3.1. Permeability Evaluation without Air–Liquid Interface (ALI)

Aerosolizing solutions of various biomolecules requires subjecting the solutions to significant agitation which may or may not result in deterioration. Therefore, mass transport of the tested biomolecules has initially been characterized in Transwells and microfluidic devices without the generation of bio-aerosols, see supplementary material.

#### 3.1.1. Permeability Measurements in Transwells

**Figure 3:**
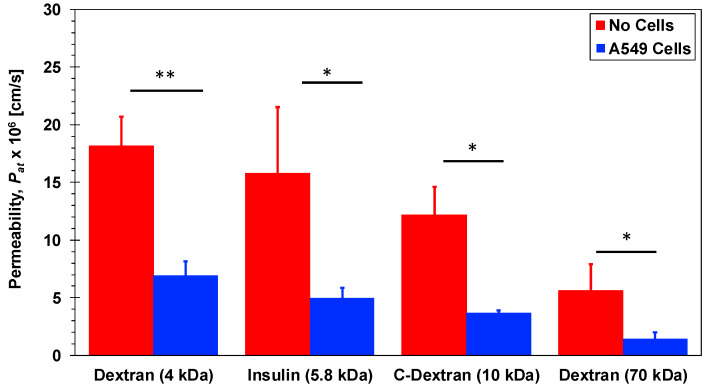
Permeability of 4-dextran, C-dextran, 70-dextran, and insulin in Transwells with and without A549 cell monolayers. Significance determined with student’s *T*-test with unequal variance (n = 3); * *p* < 0.05, ** *p* < 0.01.

Investigation of molecular transport across Transwell membranes is useful in establishing baseline permeability characteristics with no shear stress. The initial molecular concentration at the top chamber was selected to be *C*_0_ = 60, 3, 1, and 3 µM for 4-dextran, C-dextran, 70-dextran, and insulin, respectively, with zero initial concentration at the bottom chamber. The molecular concentration at the Transwell bottom chamber *C_T_* was then measured after 2 h. Molecular diffusion from the top to the bottom chamber allowing the calculation of the permeability as follows: *P_at_* = (*C_T_*/*C*_0_)(*V*/*A*)(1/*t*); where *A* = 1.12 cm^2^ is the culture area, *V* = 1500 µL is the bottom chamber volume, and *t* = 2 h is the diffusion time. The estimated permeability values for insulin and dextran molecules in Transwells, *P_at_*, are summarized in Figure 3 with and without cell monolayers. Under pure diffusion, no cells, the permeability decreases with increasing molecular size due to decreasing diffusivity. The insulin molecular size of 5.8 kDa is intermediate between the 4-dextran and the C-dextran size of 4 and 10 kDa, respectively. Accordingly, the insulin permeability of 15.8 × 10^−6^ cm/s is smaller than the 4-dextran and larger than the C-dextran permeability of 18.2 × 10^−6^ and 12.2 × 10^−6^ cm/s, respectively, while the permeability of the largest 70-dextran is the smallest at 5.6 × 10^−6^ cm/s. The presence of a confluent layer of A549 epithelial cells on the membrane, in the Transwell apical chamber, provides additional resistance to molecular diffusion through the membrane. Consequently, the permeability of each molecule type with cells is smaller than, less than half, its permeability with no cells. Dextran is typically not transported via a transcellular but via a paracellular route, which depends on the molecular size. Indeed, the transport of the largest 70-dextran is almost completely blocked by the epithelial cell layer, with permeability of about 1.4 × 10^−6^ cm/s, while the permeability of the smallest 4-dexran is the highest at about 7.0 × 10^−6^ cm/s. The decreasing permeability trend with increasing molecular size holds for the intermediate-size anionic C-dextran, 3.7 × 10^−6^ cm/s. This suggests that the ionic charge effect on the transport of C-dextran across the A549 cell layer is negligible, and its permeability is primarily determined by the molecular size. Interestingly, the permeability of insulin, 5.0 × 10^−6^ cm/s, follows the same trend as well. Unlike dextran molecules, insulin is known to be a very dynamic molecule under various circumstances [51]. Insulin at high concentration can form a hexameric crystal greatly increasing its apparent molecular size [52]. Furthermore, it has been shown that compounds insulin can form a hexamer even at low concentration (10 µM) in the presence of zinc and phenol [39]. The A549 culture media used in the experiment contains low concentrations of zinc and phenol compounds, which might give rise to the formation of insulin hexamers resulting in a significant reduction in its permeability. Since the insulin permeability was found to be larger than the C-dextran permeability, it seems that the low concentration of insulin (3 µM) combined with the small trace amounts of zinc and phenol in the culture media were not sufficient to form hexamers. Additionally, insulin has an isoelectric point at pH 5.3 suggesting it would be slightly anionic when dissolved in pH 7 culture media [53]. Similar to the C-dextran, the charge of insulin has no appreciable effect on its transport across the A549 cell layer. Hence, it is likely that under the current conditions (i.e., low concentration with no shear stress) paracellular transport of monomers is the predominant mass transfer mode of insulin across the A549 epithelial cell layer. This can serve as a reference for comparison with insulin transport in microfluidic bilayer devices under flow-induced shear stress.

#### 3.1.2. Permeability Measurements in Microfluidic Devices

**Figure 4:**
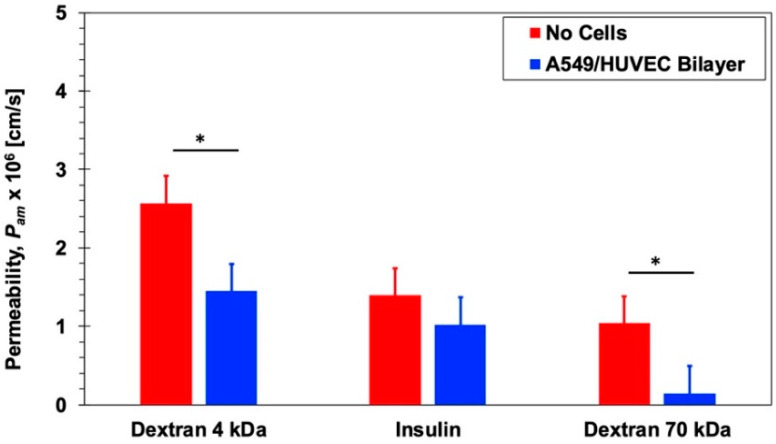
Permeability of 4-dextran, 70-dextran, and insulin in microfluidic bilayer devices with and without an epithelial/endothelial barriers. Significance determined with student’s *T*-test with unequal variance (n = 3); * *p* < 0.05.

Permeability measurements were conducted in microfluidic bilayer devices, using the 8.0 µm pore-size membrane, with and without a 549 epithelial and HUVEC endothelial cell co-culture with no air–liquid interface. The culture media was driven through each microchannel at a flow rate of *Q* = 20 µL/h resulting in flow-induced shear stress of approximately 1.2 × 10^−6^ dyne/cm^2^. Similar to the Transwell experiments, the molecular concentration at the inlet of the epithelial microchannel was *C*_0_ = 60, 1, and 3 µM for 4-dextran, 70-dextran, and insulin, respectively, with zero concentration at the inlet of the endothelial microchannel. Under steady-state conditions, following the decay of the transient response, the molecular concentration at the outlet of the endothelial microchannel *C*_∞_ was measured allowing the calculation of the permeability as follows: *P_am_* = (*C*_∞_/*C*_0_)(*Q*/*A*), where *A* = 0.2 cm^2^ is the barrier interface area. The estimated permeability values for insulin and dextran molecules in the microfluidic devices, *P_am_*, are summarized in Figure 4 with and without epithelial/endothelial barriers. The trends are qualitatively consistent with those observed in Transwells namely: (i) The permeability decreases with increasing molecular size and (ii) the presence of the epithelial/endothelial barriers decreases the permeability of all molecules. The applied shear stress due to the flowing media; therefore, has no significant effect on the characteristics of molecular transport across the membrane with and without cell barriers in microfluidic devices. In particular, paracellular transport of insulin monomers seems to remain as the prime molecular transfer mode.

### 3.2. Pharmacokinetic (PK) Analysis in Microfluidic Bilayer Devices with ALI

**Figure 5:**
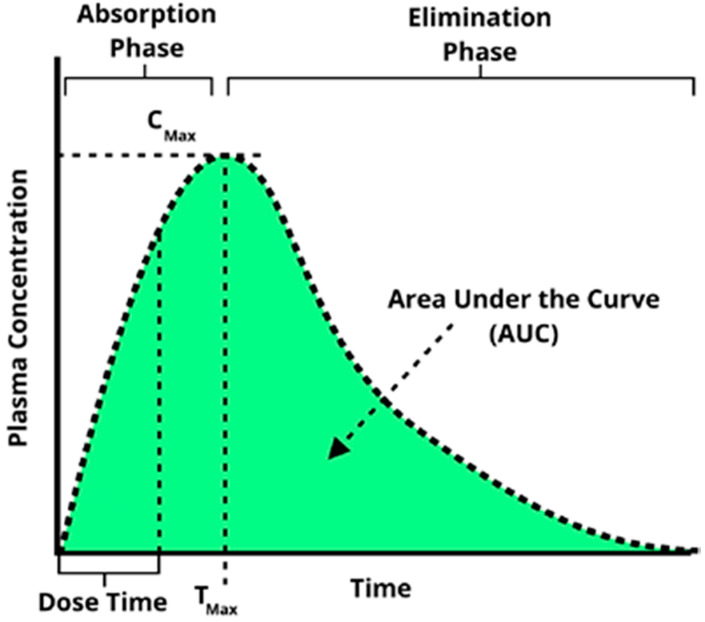
A characteristic concentration–time plot illustrating several key pharmacokinetic parameters commonly extracted from such a curve.

Pharmacokinetics is defined as the study of the time course of drug absorption, distribution, metabolism, and excretion. One of the most essential tools to evaluate adsorption for pharmacokinetic analysis is the time evolution of the concentration of a given compound in the blood stream known as the concentration–time plot. This plot is captured by continuously monitoring blood serum concentrations of a drug as a function of time using a variety of methods such as mass spectrometer or fluorescent intensity). From this plot, schematically illustrated in Figure 5, key parameters describing the behavior of the drug can be extracted including area under the concentration–time curve (AUC), maximal concentration (*C*_max_), time for maximal concentration (*T*_max_), as well as half-life (*t*_1/2_), elimination rate constant (*K_e_*), and total body clearance (*CL*). Microfluidic bilayer devices present a very attractive in vitro alternative for obtaining such concentration–time plots and, subsequently, the desired pharmacokinetic parameters.

#### 3.2.1. Construction of Concentration–Time Plots

**Figure 6:**
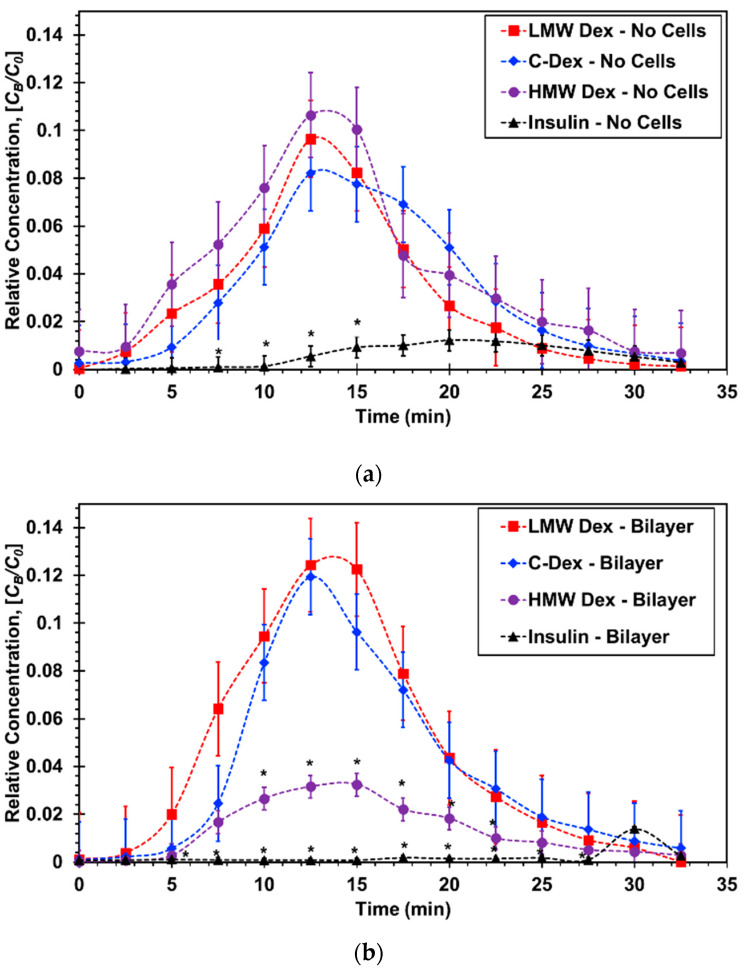
Concentration–time curves for microfluidic bilayer devices: (**a**) Without cells and (**b**) with an epithelial/endothelial barriers. * *p* < 0.05 compared with LMW Dextran.

Bio-aerosols were generated as described in Section 2.1 from molecular solutions at concentrations of *C*_0_ = 60, 1, and 3 µM for 4-dextran, 70-dextran, and insulin, respectively. The bio-aerosol, a physical mixture of air and liquid droplets, was streamed along the epithelial microchannel with and without a confluent A549 cell layer for *T*_a_ = 10 min. The driving gage pressure at the device inlet was adjusted to be 0.1 psig resulting in a flow rate of *Q* = 30 mL/min with an estimated shear stress of 2.3 dynes/cm^2^ to mimic the air flow in small airways [50,54]. The air flow was kept steady at the same rate before and after the aerosol introduction into the epithelial microchannel. The molecular concentration in the aerosol droplets is assumed to be equal to the concentration of the stock solution placed in the aerosol canister, *C*_0_, as has been reported in similar aerosol characterization studies [55]. In parallel to the aerosol flow, culture media was driven through the endothelial microchannel with and without a confluent HUVEC cell layer. A syringe pump was used to set the media flow rate at *Q* = 400 µL/h, with an estimated shear stress of 0.03 dyne/cm^2^ mimicking the blood stream and thereby establishing the air–liquid interface. As aerosol droplets accumulated on either the membrane or the A549 cell layer in the epithelial microchannel, molecules diffused from the settled droplets to the flowing media in the endothelial microchannel due to concentration gradient. The temporal molecular concentration at the outlet of the endothelial microchannel *C_B_*(*t*) was sampled at time intervals of 2.5 min allowing collection of a minimum sample volume of 15 µL needed for reliable fluorescent intensity measurement. The concentration–time plots obtained for different molecules are compared in Figure 6 with and without epithelial/endothelial barriers. The initial concentration of the stock solutions used for different molecules varied greatly to enable proper conversion of fluorescent intensity measurements to molecular concentration values using calibration curves. Therefore, in order to facilitate direct comparison between the tested molecules, the relative concentration *C_B_*/*C*_0_ was utilized for the construction of the concentration–time plots.

In devices without cells, Figure 6a, the normalized curves for the three dextran molecules of varying sizes are all very similar exhibiting the typical absorption and elimination phases. The insulin curve; however, is substantially lower indicating very limited molecular transport across the membrane. In devices with epithelial/endothelial barriers, Figure 6b, the normalized curves for the smaller dextran molecules are about the same as those with no cells suggesting that the effect of the cell barrier on the molecular transport is negligible. The largest dextran curve with cells is significantly lower than that with no cells highlighting the barrier resistance effect on the molecular transport across the epithelial/endothelial interface. More significantly, the insulin curve is practically flat with a late trace of minute absorption and elimination phases indicating that practically no insulin transport took place under the prescribed experimental conditions.

#### 3.2.2. Extraction of Pharmacokinetic Parameters

Three pharmacokinetic parameters were extracted from the concentration–time plots and normalized to render them non-dimensional as follows: (i) The area under the curve, AUC/*C*_0_*T*_a_, (ii) the maximum concentration, *C*_max_/*C*_0_, and (iii) the time to reach maximum concentration, *T*_max_/*T*_a_; where the area the area under the curve is the integral of the concentration–time plot,

(1) $$\mathrm{AUC}=\int_{0}^{\infty }C_{B}dt.$$

**Figure 7:**
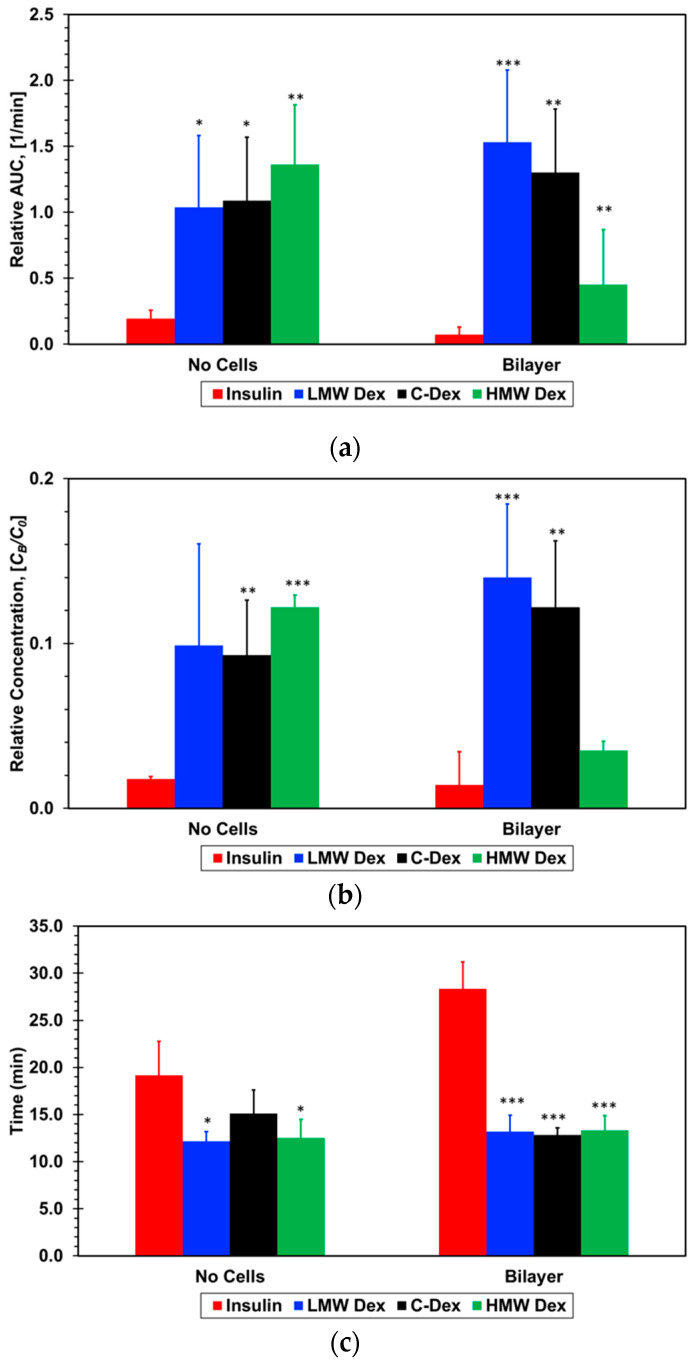
Pharmacokinetic parameters extracted from the concentration–time plots: (**a**) Relative area under the curve, AUC, (**b**) maximum concentration, *C*_max_, and (**c**) time to reach maximum concentration, *T*_max_, for the four molecules tested. * *p* < 0.05, ** *p* < 0.01, *** *p* < 0.001 compared with insulin.

The normalized PK parameters for the three dextran and insulin compounds, with and without cell barriers, are listed in Table 2 and graphed in Figure 7.

The normalized area under the curve for all three dextran compounds with no cells is about the same within experimental error, 0.12 ± 0.05, but much larger than that for insulin, 0.02 ± 0.005. With cell barriers, the normalized area for the two smaller dextran compounds is slightly higher than without cells but still within experimental error, 0.14 ± 0.05. In contrast, the area for the largest dextran is much smaller, 0.05 ± 0.02, while the area for insulin is vanishingly small, 0.007 ± 0.005. Since the area under the curve is proportional to the total amount of the absorbed compound, the presence of the epithelial/endothelial barriers had essentially no effect on the mass transport of the two smaller dextran compounds. The cell barrier had a significant effect on the paracellular transport, which is a size-dependent mechanism, of the large dextran reducing its absorbed amount to less than half the amount with no cells. It is striking that the detected amount of absorbed insulin is within the background noise level suggesting that even the trace amount absorbed with no cells is blocked by the cell barrier. While AUC represents the extent of absorption, *C*_max_ represents the rate of absorption. A direct correlation can be observed between the extracted values for the normalized maximum concentration and normalized area under the curve, with and with no cells, for all dextran and insulin compounds. This corroborates the cell barrier effects on the molecular transport delineated from the area under the curve results.

Interestingly, although the cell barrier reduced the area under the curve and maximum concentration of the large dextran to less than half of the values with no cells, there is very little difference between the small and intermediate dextran compounds. With the formation of the air–liquid interface, there is no continuous bulk flow of the molecular solution in the epithelial microchannel with a steady concentration gradient resulting in a constant molecular flux across the membrane. The only source of molecules detected in the endothelial microchannel are the droplets that accumulate on the membrane surface or cell layer in the epithelial microchannel. Hence, the amount of compound absorption depends on the droplet concentration and the number of droplets landing on the membrane surface or the epithelial cell layer rather than the molecular diffusivity. The area under the curve and the maximum concentration are normalized by the droplet concentration, and it is reasonable to assume that the aerosol droplet characteristics in terms of size and number are about the same in all experiments. Therefore, it seems that the relatively high shear stress mitigates, to a certain extent, the diffusivity effect due to molecular size, resulting in similar absorption amounts of small and intermediate dextran compounds. However, even under high shear stress, paracellular of large compounds such as 70-dextran is significantly reduced.

The time to maximum concentration, *T*_max_, is slightly longer than the aerosol flow time of *T*_a_ = 10 min varying very little, in the range of *T*_max_/*T*_a_ = 1.2–1.5, for the three dextran compounds with and without cell barriers. However, the normalized time to maximum concentration is much longer for the insulin, about 1.9 without cell barriers and 2.8 with cell barriers. This clearly indicates that insulin transport across the air–liquid interface is not a simple molecular diffusion from the settled droplets to the flowing media similar to the transport of the dextran compounds. Based on the permeability measurements in Transwells and microfluidic devices, with and without cell barriers, it was anticipated that insulin concentration–time curve would be similar to that obtained for the small Dextran since their molecular size is about the same. However, the extracted insulin PK parameters are very different from the PK values for all three dextran compounds, with and without cell barriers; both the normalized area under the curve and maximum concentration are significantly lower while the time to maximum concentration is significantly longer. This suggests that another mechanism is strongly hindering the transport of only insulin but not the transport of the dextran compounds with or without cell barriers.

#### 3.2.3. Insulin Dynamic Characterization

**Figure 8:**
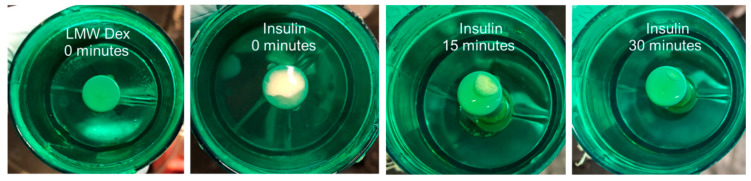
Images of the jet nebulizer platform shortly after a period of agitation for both LMW dextran and Insulin solutions as a function of time.

The substantially different concentration–time curve for insulin, in comparison with the curves for all dextran compounds, indicates that the dynamic nature of insulin plays an important role only under the air–liquid interface operation, which requires aerosol generation. The permeability results in microfluidic bilayer devices essentially preclude the formation of insulin hexamer subunits, thereby increasing its molecular size, even in the presence of zinc and phenol in the culture media under low flow-induced shear stress. Aerosolized insulin has been reported to undergo fibrillation resulting in much longer and larger chains of insulin, and problems related to insulin instability due to its agitation have been observed [56]. Insulin fibrillation has frequently been associated with the presence of an air–liquid interface [57,58,59]. In order to generate an aerosol, using a jet nebulizer, a high-pressure air stream is forced through a liquid solution to form a mixture of tiny liquid droplets suspended in air. Thus, the high stresses involved in the aerosol generation process could lead to insulin fibrillation. Visual inspection of the upper jet nebulizer platform provides an indirect evidence for insulin fibril formation. The photographs of the nebulizer platform, shown in Figure 8, were taken following 5 min of the solution agitation. A pellet, indicating solution turbidity, is visible only for insulin but not for the dextran compounds and, furthermore, the insulin pellet disappears within half an hour.

**Figure 9:**
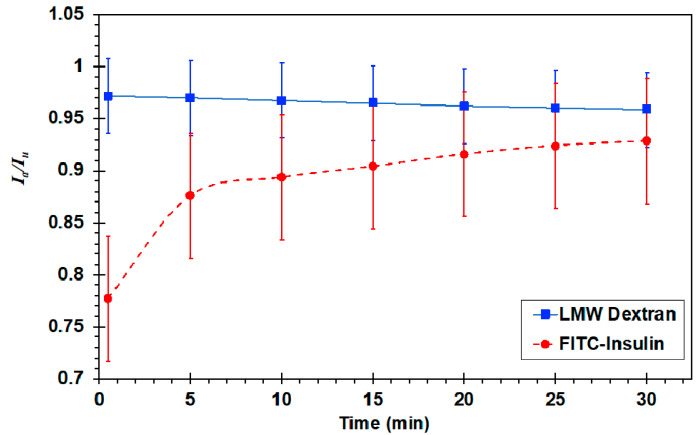
Plot of fluorescent intensity in an air–liquid agitated solution as a function of time. Final intensity refers to the solution intensity at time t = 30 min. Normalize by the intensity of un-agitated solutions, *I_a_*/*I_u_*.

The transparency of a solution represents its turbidity level, which can be quantified by measuring the light intensity of the solution with suspended fluorescently-labeled molecules [60], and the level of insulin fibril formation was reported to increase with its measured turbidity [61]. Therefore, to further support the visual inspection results, time-dependent fluorescent intensity measurements of insulin and LMW dextran solutions, *I_a_*(*t*), following 5 min agitation in a jet nebulizer, are normalized by the measured intensities of un-agitated solutions, *I_u_*, in Figure 9. The intensity of the agitated and un-agitated dextran is approximately the same (*I_a_*/*I_u_* ≅ 1), indicating constant transparency with and without agitation, which is consistent with the lack of a dextran pellet on the nebulizer platform. In contrast, the relative intensity level of the agitated insulin increases with time from an initial value of about 0.7 to a steady-state value of 1 within 20 min. The initially lower intensity of the agitated insulin with its low transparency, which is correlated with the pellet formation on the nebulizer platform, can be due to insulin fibril formation. In such a case, both the pellet visual inspection and turbidity measurement suggest that insulin fibrils dissociate within half an hour time scale back to their normal monomer structure. While the kinetics of insulin fibril dissociation depends on a variety of factors such as concentration or duration of agitation, it has indeed been shown that the dissociation process can occur fairly quickly (<1 h) under conditions similar to those encountered in the present experiments [62,63].

**Figure 10:**
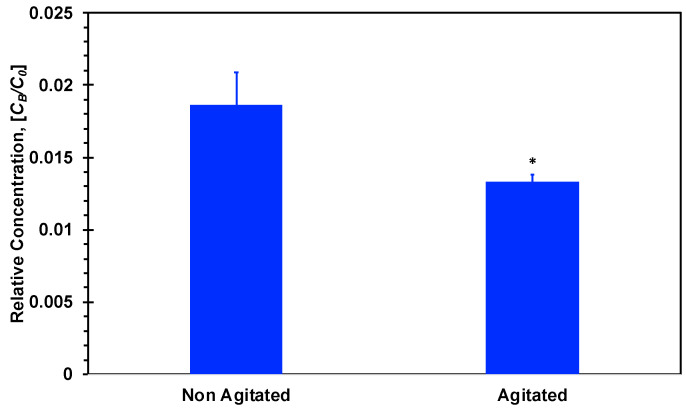
Bottom compartment relative concentration of insulin after 20 min incubation. Agitated insulin was agitated with an air–liquid interface for 5 min and then added to the upper Transwell compartment. * *p* < 0.05 compared with non-agitated insulin.

The possibility of insulin fibril formation due to its agitation during the aerosol generation process in a jet nebulizer was established. Since it is not clear whether fibril formation could affect the insulin concentration–time curve, the effect of fibril formation on insulin diffusivity was evaluated. The permeability of insulin un-agitated and agitated for 5 min in a jet nebulizer was measured in Transwells without cells only, for simplicity. The permeability values, calculated based on concentration measurements in the Transwell bottom compartments after 20 min diffusion time, are shown in Figure 10. The permeability of the agitated insulin solution is clearly lower than the permeability of the un-agitated solution suggesting that the diffusivity of the agitated insulin is lower due to its larger molecular size, which is consistent with fibril formation. Hence, this may be the cause for the substantially different PK parameters extracted for insulin in comparison with those extracted for all dextran compounds. It is; therefore, critical to maintain the stability of compounds during aerosol generation process in order to obtain reliable concentration–time plots using microfluidic bilayer devices with an air–liquid interface.

## 4. Conclusions

Pharmacokinetics and pharmacodynamics play an important role in the successful introduction of new therapeutic modalities. In comparison to current in vitro cell cultures and animal models, microfluidic bilayer devices provide superior platforms to investigate compound absorption across the human blood–air barrier, which is the first process involved in pharmacokinetic analysis. A549 epithelial and HUVEC endothelial cell barriers were established in microfluidic bilayer devices with an air–liquid interface to mimic human small airways. The transient aerosol flow in the microchannel was found to have a time scale on the order of 30 min with a median droplet diameter around 1.9 µm and about 20–25% of the droplets land in the device overlap region enabling compound absorption. The permeability of four compounds in Transwells and microfluidic devices was measured. The permeability of insulin and dextran compounds without cells in Transwells and microfluidic bilayer devices with no ALI is dominated by diffusivity, decreasing with increasing molecular size. In the presence of confluent cell layers, with negligible transcellular transport, the permeability decreases further due to decreasing paracellular transport with increasing molecular size. Consequently, the permeability of the HMW dextran, with the largest molecular size, is practically zero.

The application of microfluidic bilayer devices was extended to perform pharmacokinetic analysis of epithelial/endothelial barriers with an air–liquid interface, requiring aerosol generation, to mimic small human airways. Concentration–time curves for insulin and dextran compounds were established for the extraction of standard pharmacokinetic parameters: Area under the curve, maximum concentration, and time to maximum concentration. The cell barrier significantly affected the pharmacokinetic parameters of only the HMW dextran, as its paracellular transport was negligible due to the large molecular size. It seems that aerosolizing insulin in a jet nebulizer leads to the formation of fibrils with a substantially larger apparent molecular size. This was indirectly supported by the visual appearance of a pallet on the nebulizer platform, measured high turbidity (low transparency), as well as low diffusivity of agitated insulin; all consistent with fibril formation. Consequently, the absorbed amount of agitated insulin was too small to construct a reliable concentration–time plot. Hence, it is paramount to include stabilizing agents, such as zinc, in aerosolized compound formulations to maximize absorption across cellular barriers. In summary, the microfluidic bilayer devices incorporating different human cell types with signaling offer a distinct advantage for pharmacokinetic studies over standard 2-D cell culture or animal models.

## Figures and Tables

**Table 1:** Aerosol parameters.

Parameter	Current Experiments	Reported Values
Blood flow rate, *Q* [μL/h]	400	200–485 [43]
Aerosol exposure time, *t_exp_* [min]	10	5–30 [23,44]
Droplet size, *d* [µm]	1–15	0.5–10 [45,46]
Aerosol pressure, P [psig]	0.1	0.07 [47]
Residence time, *t_res_* [s]	0.5	0.2–3 [48]
Liquid shear stress, [dynes/cm^2^]	0.03	14 [49]
Air shear stress, [dynes/cm^2^]	2.3	2 [50]

**Table 2:** Pharmacokinetic values for microdevices.

	*AUC/C*_0_ *T*_a_	*C*_max_/*C*_0_	*T*_max_/*T*_a_
**No Cells**			
**LMW Dex**	0.10 ± 0.05	0.10 ± 0.05	1.2 ± 0.1
**C-Dex**	0.11 ± 0.05	0.09 ± 0.05	1.5 ± 0.3
**HMW Dex**	0.14 ± 0.05	0.12 ± 0.02	1.3 ± 0.2
**Insulin**	0.02 ± 0.005	0.02 ± 0.01	1.9 ± 0.4
**A549/HUVEC Bilayer**			
**LMW Dex**	0.15 ± 0.05	0.15 ± 0.05	1.3 ± 0.2
**C-Dex**	0.13 ± 0.05	0.12 ± 0.05	1.3 ± 0.1
**HMW Dex**	0.05 ± 0.02 *	0.04 ± 0.01 ***	1.4 ± 0.2
**Insulin**	0.007 ± 0.004	0.02 ± 0.01	2.8 ± 0.3 *

* *p* < 0.05, *** *p* < 0.001 for bilayer devices compared to no cell devices for the same molecule.

## Supplementary Materials

The following are available online at https://www.mdpi.com/2072-666X/11/5/536/s1, Figure S1: ImageJ generated binary images (obj: ×20) of droplets from: (a) the major aerosol outlet, and (b) in the microchannel. Figure S2: Diameter distribution profiles comparing the Jet major outlet and Jet microdevice. Vertical dashed lines represent the median diameter for each distribution and lognormal PDF’s fit to the data using a least squares method. Figure S3: Bottom channel absorption of fluorescein as a function of time exposed to aerosol. Dashed line represents a best fit approximation of the data. Figure S4: Integral of the bottom channel fluorescent intensity after 10 min of exposure to LMW-Dextran aerosol with membrane pore sizes of 0.8, 3 and 8 µm (n = 3).

## References

[1] Cuatrecasas P. (2006). Drug discovery in jeopardy. J. Clin. Investig..

[2] van der Graaf P.H., Benson N. (2011). Systems Pharmacology: Bridging Systems Biology and Pharmacokinetics-Pharmacodynamics (PKPD) in Drug Discovery and Development. Pharm. Res..

[3] Van Norman G.A. (2016). Drugs, Devices, and the FDA: Part 1. JACC: Basic Transl. Sci..

[4] DiMasi J.A., Hansen R.W., Grabowski H.G. (2003). The price of innovation: New estimates of drug development costs. J. Health Econ..

[5] DiMasi J.A., Grabowski H.G., Hansen R.W. (2016). Innovation in the pharmaceutical industry: New estimates of R&D costs. J. Health Econ..

[6] Dowden H., Munro J. (2019). Trends in clinical success rates and therapeutic focus. Nat. Rev. Drug Discov..

[7] Zhang B., Radisic M. (2017). Organ-on-a-chip devices advance to market. Lab Chip.

[8] Haddrick M., Simpson P.B. (2019). Organ-on-a-chip technology: Turning its potential for clinical benefit into reality. Drug Discov. Today.

[9] Franzen N., van Harten W.H., Retèl V.P., Loskill P., van den Eijnden-van Raaij J., IJzerman M. (2019). Impact of organ-on-a-chip technology on pharmaceutical R&D costs. Drug Discov. Today.

[10] Roth Y., Chapnik J.S., Cole P. (2003). Feasibility of Aerosol Vaccination in Humans. Ann. Otol. Rhinol. Laryngol..

[11] Laube B.L. (2014). The expanding role of aerosols in systemic drug delivery, gene therapy and vaccination: An update. Transl. Respir. Med..

[12] Stribling R., Brunette E., Liggitt D., Gaensler K., Debs R. (1992). Aerosol gene delivery in vivo. Proc. Natl. Acad. Sci. USA.

[13] Hollinger M.A. (1985). Respiratory Pharmacology and Toxicology.

[14] Dhanani J., Fraser J.F., Chan H.-K., Rello J., Cohen J., Roberts J.A. (2016). Fundamentals of aerosol therapy in critical care. Crit. Care.

[15] Agu R.U., Ugwoke M.I., Armand M., Kinget R., Verbeke N. (2001). The lung as a route for systemic delivery of therapeutic proteins and peptides. Respir. Res..

[16] Heinemann L. (2008). The Failure of Exubera: Are We Beating a Dead Horse?. J. Diabetes Sci. Technol..

[17] Benam K.H., Villenave R., Lucchesi C., Varone A., Hubeau C., Lee H.-H., Alves S.E., Salmon M., Ferrante T.C., Weaver J.C. (2016). Small airway-on-a-chip enables analysis of human lung inflammation and drug responses in vitro. Nat. Methods.

[18] Huh D., Matthews B.D., Mammoto A., Montoya-Zavala M., Hsin H.Y., Ingber D.E. (2010). Reconstituting Organ-Level Lung Functions on a Chip. Science.

[19] Huh D., Leslie D.C., Matthews B.D., Fraser J.P., Jurek S., Hamilton G.A., Thorneloe K.S., McAlexander M.A., Ingber D.E. (2012). A Human Disease Model of Drug Toxicity–Induced Pulmonary Edema in a Lung-on-a-Chip Microdevice. Sci. Transl. Med..

[20] Stucki A.O., Stucki J.D., Hall S.R.R., Felder M., Mermoud Y., Schmid R.A., Geiser T., Guenat O.T. (2015). A lung-on-a-chip array with an integrated bio-inspired respiration mechanism. Lab Chip.

[21] Frost T.S., Jiang L., Lynch R.M., Zohar Y. (2019). Permeability of Epithelial/Endothelial Barriers in Transwells and Microfluidic Bilayer Devices. Micromachines.

[22] Frost T.S., Estrada V., Jiang L., Zohar Y. (2019). Convection–Diffusion molecular transport in a microfluidic bilayer device with a porous membrane. Microfluid. Nanofluidics.

[23] Neilson L., Mankus C., Thorne D., Jackson G., DeBay J., Meredith C. (2015). Development of an in vitro cytotoxicity model for aerosol exposure using 3D reconstructed human airway tissue; application for assessment of e-cigarette aerosol. Toxicol. In Vitro.

[24] Röhm M., Carle S., Maigler F., Flamm J., Kramer V., Mavoungou C., Schmid O., Schindowski K. (2017). A comprehensive screening platform for aerosolizable protein formulations for intranasal and pulmonary drug delivery. Int. J. Pharm..

[25] Shyy J.Y.-J., Chien S. (2002). Role of Integrins in Endothelial Mechanosensing of Shear Stress. Circ. Res..

[26] Ingber D.E. (2002). Mechanical Signaling and the Cellular Response to Extracellular Matrix in Angiogenesis and Cardiovascular Physiology. Circ. Res..

[27] Mehta D., Malik A.B. (2006). Signaling Mechanisms Regulating Endothelial Permeability. Physiol. Rev..

[28] Benam K.H., Novak R., Nawroth J., Hirano-Kobayashi M., Ferrante T.C., Choe Y., Prantil-Baun R., Weaver J.C., Bahinski A., Parker K.K. (2016). Matched-Comparative Modeling of Normal and Diseased Human Airway Responses Using a Microengineered Breathing Lung Chip. Cell Syst..

[29] Bovard D., Sandoz A., Luettich K., Frentzel S., Iskandar A., Marescotti D., Trivedi K., Guedj E., Dutertre Q., Peitsch M.C. (2018). A lung/liver-on-a-chip platform for acute and chronic toxicity studies. Lab Chip.

[30] Bodier-Montagutelli E., Mayor A., Vecellio L., Respaud R., Heuzé-Vourc’h N. (2018). Designing inhaled protein therapeutics for topical lung delivery: What are the next steps?. Expert Opin. Drug Deliv..

[31] Lee K.S., Ram R.J. (2009). Plastic–PDMS bonding for high pressure hydrolytically stable active microfluidics. Lab Chip.

[32] Leslie D.C., Domansky K., Hamilton G.A., Bahinski A., Ingber D.E. Aerosol Drug Delivery for Lung on a Chip. Proceedings of the 15th International Conference on Miniaturized Systems for Chemistry and Life Sciences.

[33] Leslie D.C., Domansky K. (2014). US 20140158233 Aerosol Delivery to a Microfluidic Device. U.S. Patent.

[34] Tan S.N., Pugh R.J., Fornasiero D., Sedev R., Ralston J. (2005). Foaming of polypropylene glycols and glycol/MIBC mixtures. Miner. Eng..

[35] Yuan H., Miao J., Du Y.-Z., You J., Hu F.-Q., Zeng S. (2008). Cellular uptake of solid lipid nanoparticles and cytotoxicity of encapsulated paclitaxel in A549 cancer cells. Int. J. Pharm..

[36] Quigg R.J., Cybulsky A.V., Jacobs J.B., Salant D.J. (1988). Anti-Fx1A produces complement-dependent cytotoxicity of glomerular epithelial cells. Kidney Int..

[37] Grudzielanek S., Velkova A., Shukla A., Smirnovas V., Tatarek-Nossol M., Rehage H., Kapurniotu A., Winter R. (2007). Cytotoxicity of Insulin within its Self-assembly and Amyloidogenic Pathways. J. Mol. Biol..

[38] Wang S.S.-S., Liu K.-N., Han T.-C. (2010). Amyloid fibrillation and cytotoxicity of insulin are inhibited by the amphiphilic surfactants. Biochim. Biophys. Acta Mol. Basis Dis..

[39] Gast K., Schüler A., Wolff M., Thalhammer A., Berchtold H., Nagel N., Lenherr G., Hauck G., Seckler R. (2017). Rapid-Acting and Human Insulins: Hexamer Dissociation Kinetics upon Dilution of the Pharmaceutical Formulation. Pharm. Res..

[40] Muzaffar M., Ahmad A. (2011). The Mechanism of Enhanced Insulin Amyloid Fibril Formation by NaCl Is Better Explained by a Conformational Change Model. PLoS ONE.

[41] Moraes C., Labuz J.M., Leung B.M., Inoue M., Chun T.-H., Takayama S. (2013). On being the right size: Scaling effects in designing a human-on-a-chip. Integr. Biol..

[42] Wikswo J.P., Curtis E.L., Eagleton Z.E., Evans B.C., Kole A., Hofmeister L.H., Matloff W.J. (2013). Scaling and systems biology for integrating multiple organs-on-a-chip. Lab Chip.

[43] Singhal S., Henderson R., Horsfield K., Harding K., Cumming G. (1973). Morphometry of the Human Pulmonary Arterial Tree. Circ. Res..

[44] Gardenhire D.S., Burnett D., Strickland S., Myers T.R. (2017). Aerosol Delivery Devices for Respiratory Therapists.

[45] Laube B.L., Janssens H.M., de Jongh F.H.C., Devadason S.G., Dhand R., Diot P., Everard M.L., Horvath I., Navalesi P., Voshaar T. (2011). What the pulmonary specialist should know about the new inhalation therapies. Eur. Respir. J..

[46] Patil-Gadhe A., Kyadarkunte A., Patole M., Pokharkar V. (2014). Montelukast-loaded nanostructured lipid carriers: Part II Pulmonary drug delivery and in vitro–in vivo aerosol performance. Eur. J. Pharm. Biopharm..

[47] Levitzky M.G. (2013). Chapter 2. Mechanics of Breathing. Pulmonary Physiology.

[48] Khajeh-Hosseini-Dalasm N., Longest P.W. (2015). Deposition of particles in the alveolar airways: Inhalation and breath-hold with pharmaceutical aerosols. J. Aerosol Sci..

[49] Tang B.T., Pickard S.S., Chan F.P., Tsao P.S., Taylor C.A., Feinstein J.A. (2012). Wall Shear Stress is Decreased in the Pulmonary Arteries of Patients with Pulmonary Arterial Hypertension: An Image-Based, Computational Fluid Dynamics Study. Pulm. Circ..

[50] Nucci G., Suki B., Lutchen K. (2003). Modeling airflow-related shear stress during heterogeneous constriction and mechanical ventilation. J. Appl. Physiol..

[51] Attri A.K., Fernández C., Minton A.P. (2010). pH-dependent self-association of zinc-free insulin characterized by concentration-gradient static light scattering. Biophys. Chem..

[52] Hassiepen U., Federwisch M., Mülders T., Wollmer A. (1999). The Lifetime of Insulin Hexamers. Biophys. J..

[53] Wintersteiner O., Abramson H.A. (1933). The Isolectric Point of Insulin. J. Biol. Chem..

[54] Sul B., Wallqvist A., Morris M.J., Reifman J., Rakesh V. (2014). A computational study of the respiratory airflow characteristics in normal and obstructed human airways. Comput. Biol. Med..

[55] Stapleton K.W., Finlay W.H. (1995). Determining solution concentration within aerosol droplets output by jet nebulizers. J. Aerosol Sci..

[56] Vinther T.N., Norrman M., Ribel U., Huus K., Schlein M., Steensgaard D.B., Pedersen T.Å., Pettersson I., Ludvigsen S., Kjeldsen T. (2013). Insulin analog with additional disulfide bond has increased stability and preserved activity: Insulin Analog with Additional Disulfide Bond. Protein Sci..

[57] Nielsen L., Khurana R., Coats A., Frokjaer S., Brange J., Vyas S., Uversky V.N., Fink A.L. (2001). Effect of Environmental Factors on the Kinetics of Insulin Fibril Formation: Elucidation of the Molecular Mechanism. Biochemistry.

[58] Sluzky V., Tamada J.A., Klibanov A.M., Langer R. (1991). Kinetics of insulin aggregation in aqueous solutions upon agitation in the presence of hydrophobic surfaces. Proc. Natl. Acad. Sci. USA.

[59] Sluzky V., Klibanov A.M., Langer R. (1992). Mechanism of insulin aggregation and stabilization in agitated aqueous solutions. Biotechnol. Bioeng..

[60] Biswal N., Gupta S., Ghosh N., Pradhan A. (2003). Recovery of turbidity free fluorescence from measured fluorescence: An experimental approach. Opt. Express.

[61] Jarrett J.T., Lansbury P.T. (1992). Amyloid fibril formation requires a chemically discriminating nucleation event: Studies of an amyloidogenic sequence from the bacterial protein OsmB. Biochemistry.

[62] Shammas S.L., Knowles T.P.J., Baldwin A.J., MacPhee C.E., Welland M.E., Dobson C.M., Devlin G.L. (2011). Perturbation of the Stability of Amyloid Fibrils through Alteration of Electrostatic Interactions. Biophys. J..

[63] Mishra R., Winter R. (2008). Cold- and Pressure-Induced Dissociation of Protein Aggregates and Amyloid Fibrils. Angew. Chem. Int. Ed..

