# Supplementary material for: Pharmacokinetic Analysis of Epithelial/Endothelial Cell Barriers in Microfluidic Bilayer Devices with an Air–Liquid Interface
Source: Micromachines (Basel). 2020 May 25;11(5):536. doi: 10.3390/mi11050536 (PMC7281310; doi:10.3390/mi11050536)
Supplement: Supplementary file 1 [file micromachines-11-00536-s001.pdf]

micromachines

MDPI

Supplemental Information

S1 Droplet size and spatial distribution

A major consideration in the development of an aerosolized therapeutic is the droplet size desired for optimum compound absorption into the bloodstream. Different droplet diameters for an aerosolized therapy can affect compound absorption across the lung-blood barrier [1,2] and, subsequently, influence the treatment efficacy [3,4]. Ideal droplet size for absorption in the deeper pulmonary regions has been reported to be in the range of 0.5–10 µm [1,5]. Following similar droplet analysis studies [6], the droplet size was evaluated using fluorescent microscopy and image processing. The apparatus inlet pressure was adjusted to maintain aerosol flow under constant device inlet pressure of 0.1 psig. The aerosol flow along the microchannel was kept steady for 10 s and, simultaneously, a glass microscope slide was exposed to the stream at the major aerosol outlet. Droplets collected in the microchannel and on the glass slide were imaged under a fluorescent microscope as shown in Figure S1.

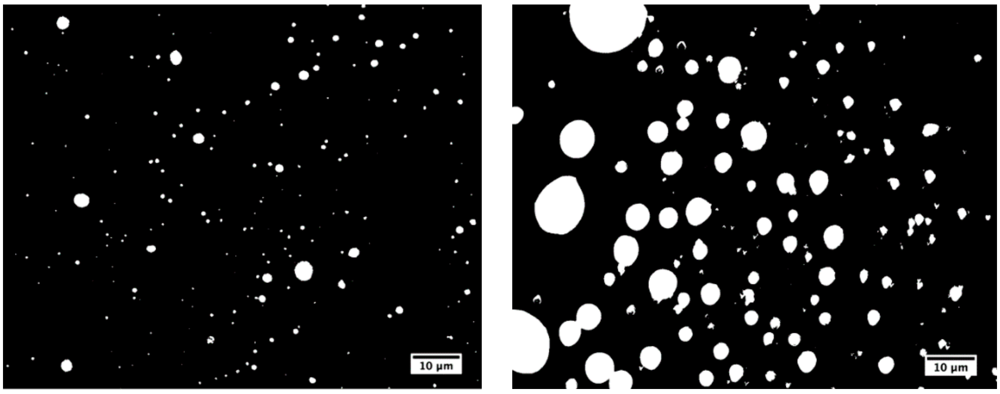

(**a**) (**b**)

Figure S1. ImageJ generated binary images (obj: ×20) of droplets from: (**a**) the major aerosol outlet, and (**b**) in the microchannel.

The recorded images were processed using ImageJ (NIH) to construct the droplet size histograms plotted in Figure S2. The droplet diameter is a positive-definite variable; hence, the droplet size histogram was fitted with a lognormal probability distribution function:

$$P(d) = \frac{1}{d\sigma\sqrt{2\pi}} exp\left(-\frac{(ln(d)-\mu)^2}{2\sigma^2}\right) \quad (1)$$

where d is the droplet diameter, σ is the standard deviation, and μ is the natural logarithmic mean for the data. Since the droplet distributions were fitted by log-normal rather than normal probability distribution function (PDF), the Wilcoxon-rank sum test was used for hypothesis testing instead of a student’s t-test. The median diameter of droplets collected at the major aerosol outlet using the jet nebulizer was about 1.0 µm. The size of droplets collected inside the device, from the minor aerosol outlet, was larger with a median diameter around 1.9 µm ($p < 0.05$). This suggests that droplets tend to coagulate as they enter the microchannel increasing their average size, and coagulation of aerosols in tubes has been experimentally investigated [7].

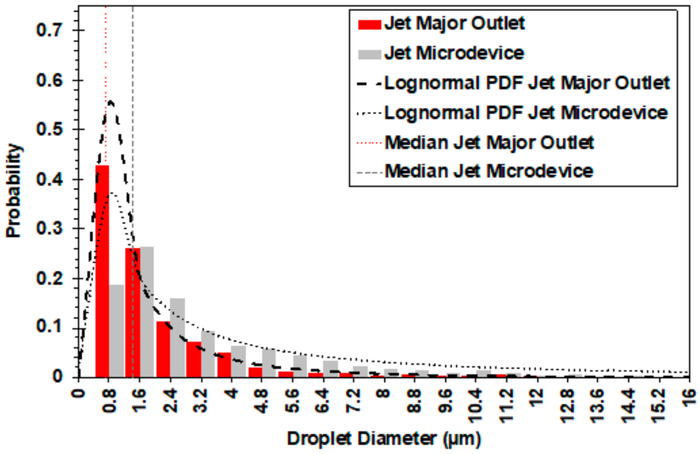

Figure S2. Diameter distribution profiles comparing the Jet major outlet and Jet microdevice. Vertical dashed lines represent the median diameter for each distribution and lognormal PDF's fit to the data using a least squares method.

Droplet placement along a microchannel is also important for uniform exposure of the epithelium to the aerosol stream along the segment where compound absorption occurs. To assess this, a fluorescein solution was placed in the aerosol canister, and the device was connected to the aerosol flow for 10 min under a constant inlet pressure of 0.1 psig. The device was then disconnected from aerosol flow, and the microchannel was viewed with a fluorescent microscope to determine both the size and location of droplets. Major droplet aggregation sites were observed around the microchannel inlet and outlet as well as near the bends. These regions represent areas of higher shear stress associated with changes in flow direction. Although the aerosol flow in essentially the laminar, droplet deposition is still heavily influenced by the channel geometry similar to the more complex geometry observed throughout a lung [8]. The probability for a droplet to land in a region where both channels overlap allowing compound absorption was estimated to be about 20–25%.

S2 Transient aerosol flow

The transient performance of the microfluidic device without cells was characterized under a constant aerosol flow. The aerosol canister in the jet nebulizer was fed by a dedicated syringe pump with a fluorescein solution at a constant rate of 15 mL/hr. The minor aerosol flow was driven through the device epithelial microchannel with a constant inlet pressure of 0.1 psig, while media was passed through the device endothelial microchannel at a constant flow rate of 400 µL/hr. This flow pattern was maintained for 30 min, which is longer than the dosage time typically used in pharmacokinetic analysis. Samples of the media flow carrying the absorbed fluorescein were collected from the endothelial channel outlet every 2.5 min, each about 15 µL in volume, to measure the time dependent light intensity level using the plate reader. The corresponding fluorescein concentration is depicted in Figure S3 as a function of time. The fluorescein concentration initially increased almost linearly for about 15 min, after which the concentration leveled off but without the establishment of a clear steady-state. This could be due to the interaction between the aerosol droplets and the solid boundary along the aerosol stream.

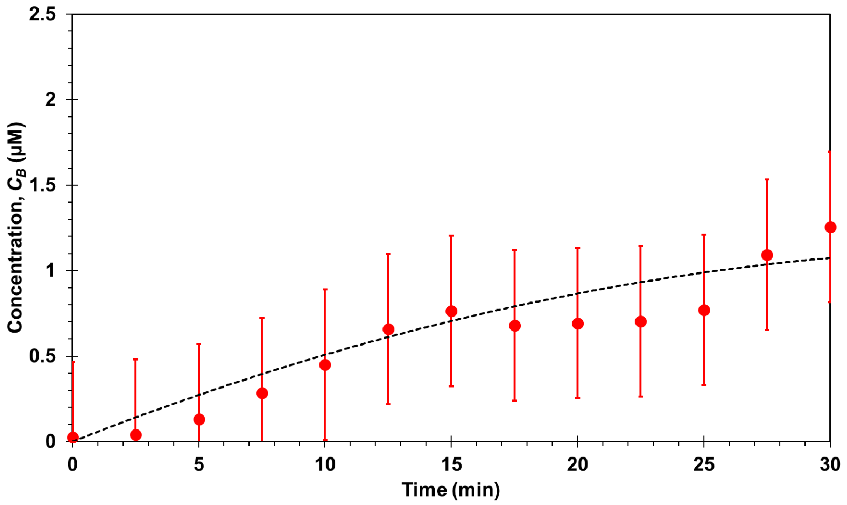

Figure S3. Bottom channel absorption of fluorescein as a function of time exposed to aerosol. Dashed line represents a best fit approximation of the data.

S3 Pore-size and pressure effects

A major component of a microfluidic bilayer device used for modeling a tissue-level complexity is the porous membrane allowing co-cultivation of different cell types [9]. The amount of compound transported across the membrane depends on the aerosol flow rate, controlled by the aerosol channel inlet pressure, and membrane porosity, determined by the pore size and number of pores. Therefore, membranes varying in pore size were tested under different inlet pressures. A two-factor multi-level ANOVA experiment was used to assess the porosity and pressure effects on aerosol absorption into the bottom liquid stream. Experiments were conducted with membranes having a pore diameter of 0.8, 3.0, and 8.0 µm under aerosol inlet pressure of 0.05, 0.1, and 0.2 psig. A constant aerosol flow of LMW Dextran was kept for 10 min along the epithelial microchannel with constant media flow rate of 400 µL/hr along the endothelial microchannel. Samples of the endothelial channel outlet flow were collected to measure the LMW Dextran concentration, and the results are summarized in Figure S4.

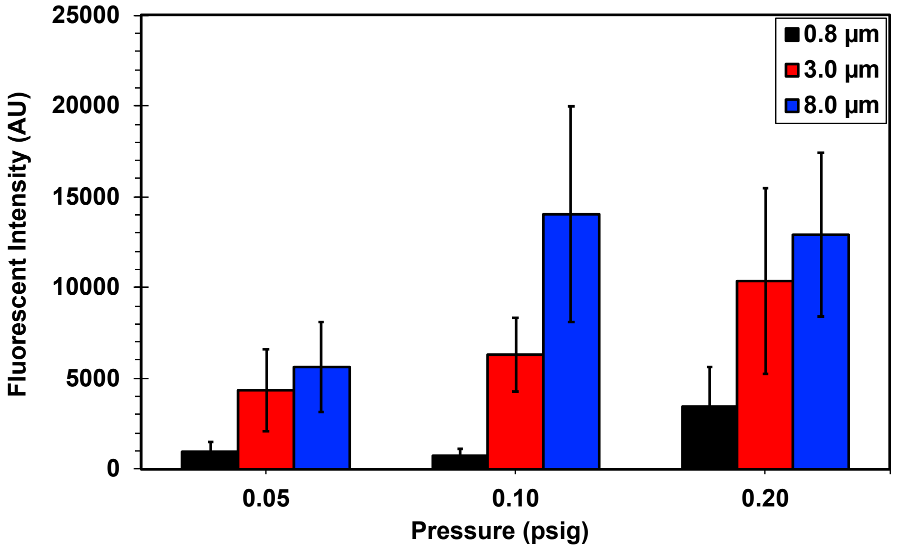

Figure S4. Integral of the bottom channel fluorescent intensity after 10 min of exposure to LMW-Dextran aerosol with membrane pore sizes of 0.8, 3 and 8 µm (n = 3).

In general, increasing the aerosol inlet pressure and flow rate results in increasing compound absorption. As has been previously reported [10], the amount of aerosol droplets retained by the membrane increases with increasing average aerosol velocity in the channel due to a higher flow rate.

Consequently, the accumulation of more droplets on the membrane would result in a higher compound absorption rate. Increasing transmembrane pressure could also play a minor role in enhancing the compound transmembrane absorption [11]. The results indicate that compound absorption also increases with increasing pore size. The membrane with the smallest pore diameter of 0.8 μm has the lowest porosity of 1%. Therefore, its compound absorption across this membrane is expected to be the lowest having the smallest diffusion area due to the small porosity. The small pore size could also affect marginally the compound transport from the droplets accumulated on the membrane to the media flow. However, although the 2.5% porosity of the 8.0 μm membrane is lower than the 10% porosity of the 3.0 μm membrane, the compound absorption measured for the 8.0 μm membrane is higher. This could be partially due to the large experimental error in measuring relatively low concentration and perhaps affected by the droplets interacting with pores spatially-distributed randomly as reported elsewhere [12]. Ultimately as a result of this higher absorption, the 8.0 μm membrane was used for all other aerosol absorption experiments using the microfluidic bilayer devices.

References

1. Labiris, N.R.; Dolovich, M.B. Pulmonary drug delivery. Part I: Physiological factors affecting therapeutic effectiveness of aerosolized medications: Physiological factors affecting the effectiveness of inhaled drugs. *Br. J. Clin. Pharmacol.* **2003**, *56*, 588–599, doi:10.1046/j.1365-2125.2003.01892.x.
2. Bochner, B.S.; Burks, A.W.; Busse, W.W.; Holgate, S.T.; Lemanske, R.F.; O'Hehir, R.E.; Adkinson, N.F.; Middleton, E. *Middleton's Allergy: Principles and Practice*; 8th ed.; Adkinson, N.F., Jr., Bochner, B.S., Burks, A.W., Busse, W.W., Holgate, S.T., Lemanske, R.F., O'Hehir, R.E., Eds.; Elsevier/Saunders: Philadelphia, PA, USA, 2014.
3. Zanen, P.; Go, L.T.; Lammers, J.W. Optimal particle size for beta 2 agonist and anticholinergic aerosols in patients with severe airflow obstruction. *Thorax* **1996**, *51*, 977–980, doi:10.1136/thx.51.10.977.
4. Zanen, P.; Go, L.T.; Lammers, J.W.J. The optimal particle size for β-adrenergic aerosols in mild asthmatics. *Int. J. Pharm.* **1994**, *107*, 211–217.
5. Khajeh-Hosseini-Dalasm, N.; Longest, P.W. Deposition of particles in the alveolar airways: Inhalation and breath-hold with pharmaceutical aerosols. *J. Aerosol Sci.* **2015**, *79*, 15–30, doi:10.1016/j.jaerosci.2014.09.003.
6. van Dalen, G. Determination of the water droplet size distribution of fat spreads using confocal scanning laser microscopy. *J. Microsc.* **2002**, *208*, 116–133, doi:10.1046/j.1365-2818.2002.01075.x.
7. Gubaidullin, D.A.; Zaripov, R.G.; Galiullin, R.G.; Galiullina, E.R.; Tkachenko, L.A. An Experimental Investigation of the Coagulation of Aerosol in a Tube in the Vicinity of Subharmonic Resonance. *High Temp.* **2004**, *42*, 794–802, doi:10.1023/B:HITE.0000046678.95056.cc.
8. Carvalho, T.C.; Peters, J.I.; Williams III, R.O. Influence of particle size on regional lung deposition—What evidence is there? *Int. J. Pharm.* **2011**, *406*, 1–10, doi:10.1016/j.ijpharm.2010.12.040.
9. Arık, Y.B.; van der Helm, M.W.; Odijk, M.; Segerink, L.I.; Passier, R.; van den Berg, A.; van der Meer, A.D. Barriers-on-chips: Measurement of barrier function of tissues in organs-on-chips. *Biomicrofluidics* **2018**, *12*, 042218, doi:10.1063/1.5023041.
10. Spurny, K.; Lodge, J.P.; Frank, E.R.; Sheesley, D.C. Aerosol filtration by means of Nuclepore filters: Aerosol sampling and measurement. *Environ. Sci. Technol.* **1969**, *3*, 464–468, doi:10.1021/es60028a005.
11. Gradoń, L.; Orlicki, D.; Podgórski, A. Deposition and Retention of Ultrafine Aerosol Particles in the Human Respiratory System. Normal and Pathological Cases. *Int. J. Occup. Saf. Ergon.* **2000**, *6*, 189–207, doi:10.1080/10803548.2000.11076451.
12. Smith, T.N.; Phillips, C.R. Inertial collection of aerosol particles at circular aperture. *Environ. Sci. Technol.* **1975**, *9*, 564–568, doi:10.1021/es60104a009.
